# The accuracy of non-contrast brain CT scan in predicting the presence of a vascular etiology in patients with primary intracranial hemorrhage

**DOI:** 10.1038/s41598-023-36042-2

**Published:** 2023-06-09

**Authors:** Bita Abbasi, Raheleh Ganjali, Reza Akhavan, Ahmadreza Tavassoli, Fatemeh Khojasteh

**Affiliations:** 1 Department of Radiology, Faculty of Medicine, Mashhad University of Medical Sciences, Mashhad, Iran; 2 Department of Medical Informatics, Faculty of Medicine, Mashhad University of Medical Sciences, Mashhad, Iran; 3 Clinical Research Development Unit, Imam Reza Hospital, Mashhad University of Medical Sciences, Mashhad, Iran; 4 Department of Emergency Medicine, Faculty of Medicine, Mashhad University of Medical Sciences, Mashhad, Iran

**Keywords:** Cerebrovascular disorders, Brain imaging, Computed tomography

## Abstract

Spontaneous intraparenchymal cerebral hemorrhages (SIPH) account for 10–15% of acute strokes. Sorting these patients according to the risk of harboring an underlying vascular etiology may help selecting the patients who would mostly benefit from Multidetector CT Angiography (MDCTA). The aim of this study was to evaluate the accuracy of Non-Contrast brain CT (NCCT) in predicting possible vascular etiologies in patients with SIPH. In this retrospective study, we evaluated the NCCT of 334 patients who presented with SIPH from March 2017 to March 2021 and we looked for vascular etiologies in the CTA which was performed for these patients. We used NCCT criteria to predict the presence of any vascular etiologies in SIPH patients and proposed a scoring system based on these criteria which might predict the risk of vascular ICH (VICH score). Out of 334 evaluated patients, 9.3% had an underlying vascular etiology. Independent predictors of the vascular etiology included: age < 46 years, no history of hypertension and coagulation disorders, lobar hemorrhages, and presence of significant perilesional edema. We used these criteria and NCCT classification to create a practical scoring system to predict the risk of vascular ICH (VICH). In our study, VICH score ≥ 4 had 51.6% sensitivity and 96.4% specificity for predicting a positive MDCTA as the maximum optimal cut-off point. The VICH score seemed to be successful in predicting vascular etiologies in this retrospective cohort of 334 patients. This scoring system can be used to select patients if there are limited resources to perform CT angiography.

## Introduction

Spontaneous intraparenchymal hemorrhages (ICH) are nontraumatic hemorrhages affecting the brain parenchyma and account for 10–15% of cases of acute stroke^1–3^. Although most cases of ICH are caused by hypertension, amyloid angiopathy, or impaired coagulation, many occur because of aneurysms, arteriovenous malformations (AVM), Dural vein thrombosis and Dural Arteriovenous Fistulas (AVF)^3,4^.

Using NCCT signs in patients with ICH, alone or accompanied by clinical variables on the triage and monitoring of patients with ICH is desirable, particularly when there are low-resource settings or interpreting the CTA is challenging. Proper management of ICH depends mainly on finding the etiology of the bleeding, especially in cases where surgical or endovascular intervention reduces the risk of bleeding^5^.

Digital catheter angiography (DSA) as the modality of choice for the diagnosis of vascular abnormalities is being largely replaced by computed tomography angiography (CTA) as the standard of diagnosis^6^. DSA is an invasive procedure that may have some potential risks of causing neurologic deficits^7^.

Also, CTA offers several advantages over conventional angiography. It is an available, rapid (only a few minutes using a multidetector row CT), and non-invasive modality that is more applicable in critically ill patients. It is more cost-effective than conventional angiography because of its lower cost, favorable risk profile, and high sensitivity in detecting vascular lesions^8–10^. However, there are some troubles with CTA. One is an added radiation dose of approximately 2.5 mSV after the NCCT which is also 2.5 mSV (total of 5 mSV). Another is that intravenous contrast agents may probably cause contrast nephropathy, especially in patients with a history of nephropathy^11^.

It is of good practice to have clinical and imaging criteria for selecting the patients who would benefit most from CTA, especially if there are limited resources for CTA and/or DSA.

Previous studies have identified clinical and non-contrast computed tomography (NCCT) features such as younger age, neither known hypertension nor impaired coagulation, presence of subarachnoid or intraventricular hemorrhage^12,13^, and temporal or frontal lobe location^14^, which are associated with a higher detection rate of vascular abnormalities on CTA or DSA.

To date, there are limited studies about the stratification of patients with ICH according to the risk of harboring an underlying vascular etiology. The purpose of this study is to evaluate the diagnostic accuracy of NCCT findings in detecting patients with an underlying vascular etiology of ICH.

## Methods

### Patient selection

The ethics committee of Mashhad university of medical sciences approved the study (approval code: IR.MUMS.MEDICAL.REC.1400.097) and waived the need for informed consent.

We searched our Picture Archiving and Communicating System (PACS) of our tertiary-level academic hospital between 2017 and 2021 for four years and collected all consecutive patients hospitalized for intra-parenchymal brain hemorrhage. Inclusion criteria were patients with acute neurologic symptoms who were diagnosed with ICH in the non-contrast brain CT scan (NCCT) at presentation, age above eighteen years, and available brain CTA obtained within 48 h from primary NCCT. The exclusion criteria were history of head trauma within the previous two weeks, evidence of ischemic stroke on the site of hemorrhage, evidence of aneurysmal hemorrhage in the CTA, history of known vascular malformation or vascular mass within the brain, known amyloid angiopathy according to Boston’s criteria, and the presence of severe artifacts in NCCT or CTA making the interpretation challenging and incomplete imaging protocol. A total of 334 patients were enrolled in this study.

### Imaging

All MDCTA and NCCT examinations were performed with a commercially available 16-MDCT scanner (Neusoft, Neuviz 16). NCCT was performed in a head holder by an axial technique with 120 kilovolts (peak), 150 mA, and 5-mm thickness reconstruction. MDCTA was performed by scanning from the base of the C1 body to the vertex using the following parameters: pitch (1.2); collimation, 1.25 mm; maximal mA, 250; kilovolt (peak), 120; FOV, 22 cm; and 100 mL of iodinated contrast material (Iodixanol 320 mg/100 mL) with the flow rate of 4 mL/s, followed by 50 mL of saline chaser injected with the same flow rate into the antecubital vein with a 25-s delay between starting the contrast injection and the start of scanning.

#### Image interpretation

Two radiologists, including an interventional radiologist with 10 years of experience in vascular imaging, and a general radiologist with 4 years of experience in general radiology, assessed the non-contrast CT scans and MDCTA images. In cases of discrepancy, a third opinion was sought from another radiologist with 5 years of experience. Image interpretation was performed on a standard PACS workstation. They were asked to record the location of ICH (lobar, deep grey matter or pons, and infratentorial), the presence of intraventricular hemorrhage (IVH), or subarachnoid hemorrhage (SAH) and the presence of considerable perilesional edema in NCCTs.

**Figure 1:**
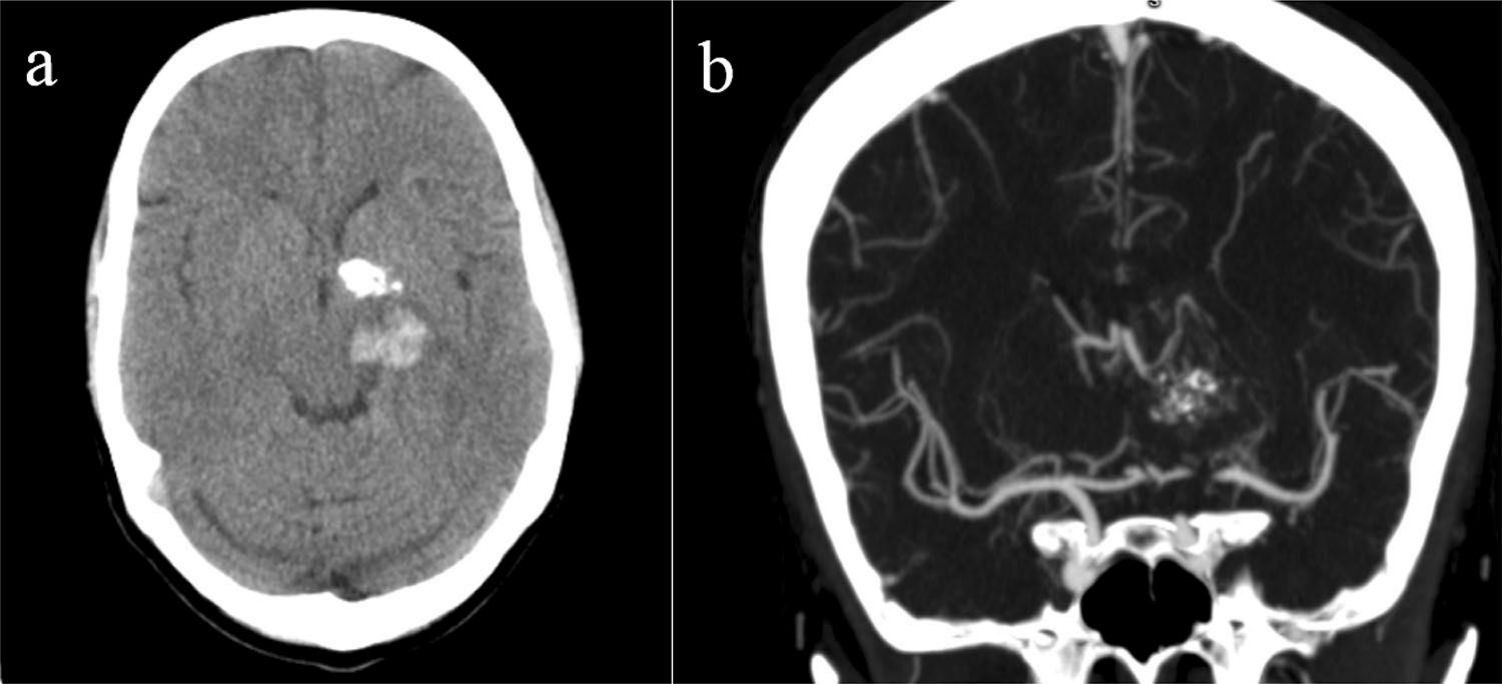
Axial non contrast CT scan (**a**) shows left basal ganglia hemorrhage and a round calcified lesion near it. The findings are high probable of underlying vascular lesion and showed to be an Arteriovenous malformation (AVM) in brain CTA (**b**).

They also evaluated the probability of underlying vascular lesions into three categories of high probable, indeterminate, and low probable according to the following criteria used in the previous literature ^10^:

High probable: The presence of enlarged vessels, with associated calcifications around the lesion or increased dural vein attenuation. (Fig. 1).

**Figure 2:**
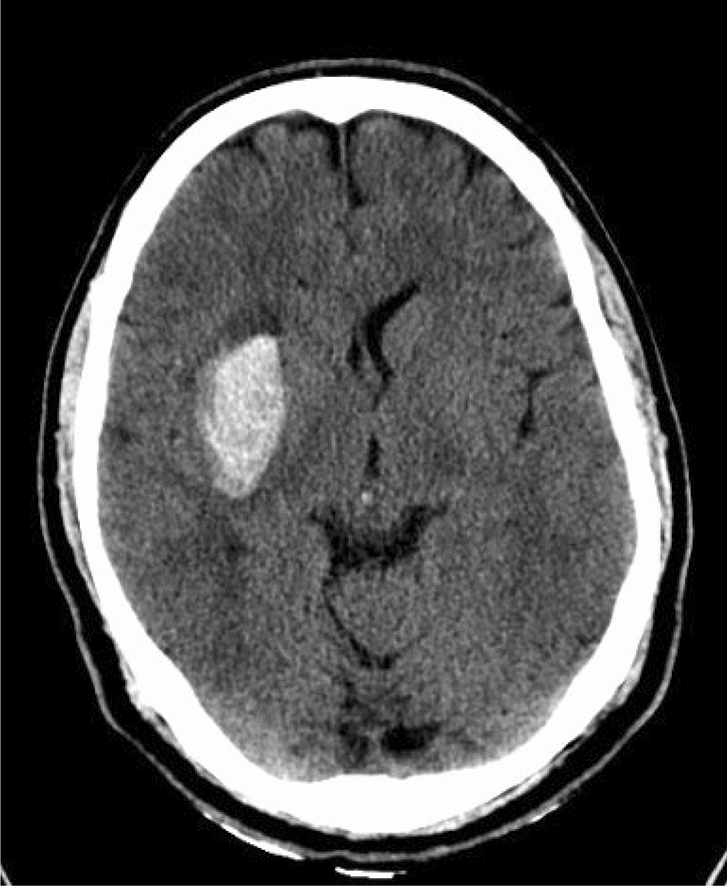
39-year-old man with history of hypertension. Axial non contrast CT scan shows right basal ganglia hemorrhage with surrounding edema. These findings are low probable of underlying vascular lesions and no vascular etiology was found on brain CTA.

Low probable: ICH in the pons or deep grey matter, without associated high-probable criteria. (Fig. 2).

Indeterminate: the lesions which do not fall in either of the above criteria.

CTA images were then interpreted by the same radiologists. The CTA images were evaluated at least two weeks after the NCCT image to prevent recall bias. The final CTA outcome was recorded as positive/negative according to the presence of vascular lesions (AVM, dural AVF, dural vein thrombosis, etc.) in the CTA images.

Other diagnostic data including MRA/MRV results, surgical and pathological reports, and digital subtraction angiography (DSA), etc. were also recorded. If other diagnostic methods revealed a vascular lesion not detected by CTA, the final analysis included the results.

Furthermore, we used the independent predictors of a positive CTA (NCCT probability, age, hypertension, impaired coagulation, IVH or SAH, location of ICH, associated edema), to construct a practical scoring system to predict the risk of vascular etiology in ICH patients, so called the Vascular ICH score (VICH score).

### Medical record review

Medical records were reviewed for patient age, sex, presence of known hypertension, and presence of coagulopathy. We divided our patients according to their age into one of the following two categories: group 1, 18–45 years of age; and group 2, patients 46 years of age and older.

Patients were also classified as hypertensive if they had a history of hypertension on medical records or were taking antihypertensive medications at presentation. Patients were classified as having coagulopathy if, at presentation, they were receiving daily anti-platelet therapy with aspirin (at least 81 mg) and/or clopidogrel had a platelet count of < 50,000 cells per cubic millimeter of blood, were on anticoagulation with warfarin and had an international normalization ratio (INR) > 1.5, or were on anticoagulation with heparin and had an active partial thromboplastin time (aPTT) of > 80 s.

### Statistical analysis

All the obtained data were collected on a database. Demographic, historical and clinical characteristics are summarized using descriptive statistics. A comparison between the groups.was made by conducting T tests. Categorical variables were compared using the χ2 test. Multivariable logistic regression models were conducted to investigate the association between the VICH scores and positive CTAs. Data were analyzed using IBM SPSS version 26. A p-value of less than 0.05 was considered statistically significant.

### Ethical considerations

The ethics committee of Mashhad university of medical sciences approved the study (approval code: IR.MUMS.MEDICAL.REC.1400.097) and waived the need for informed consent.

Patients' personal information, including names, was removed from the images and was replaced with a code unique to every individual. Patients’ medical and personal information were not shared outside the research group.

## Results

### Patient characteristics

From March 2017 to March 2021 a total of 385 patients presented to our emergency department with ICH on NCCT and were further evaluated with MDCTA of the brain within 48 h of presentation. Fifty-one patients were excluded because of the presence of an intracranial aneurysm in the CTA, and a total of 334 patients were analyzed. The mean age of patients was 54.25 (range, 18–87) years, 204 (61.1%) of whom were men, and 130 (38.9%) were women. The demographic characteristics of patients are summarized in Table 1.

**Table 1:** Demographic characteristics of 385 patients presented with acute Intracerebral hemorrhage (ICH).

Characteristic	Gender		Total(N = 334)	*p*-value
	Male(N = 204, 61.1%)	Female (N = 130, 38.9%)		
Age, median (IQR)	54 (43–65)	54 (25–75)	54 (45–64)	0.89*
Hypertension, number (%)				
No	67 (32.8%)	37 (28.5%)	104 (31.1%)	0.47**
Yes	137 (67.2%)	93 (71.5%)	230 (68.9%)	
Coagulopathy, number (%)***				
No	196 (96.1%)	125 (96.2%)	321 (96.1%)	0.77**
Yes	8 (3.9%)	4 (3.1%)	12 (3.6%)	

*Independent T-Test; **Chi square test, ***There was 1 missing data.

The patients were categorized according to their age into the two groups of 45 years or younger, and older than 45 years. The vascular etiology was found in 19.8% of patients 45 years or younger and in 5.3% of patients older than 46 years old (*p*-value < 0.000).

### Vascular ICH etiologies in the patient population

Thirty-one patients (9.3%) out of a total of 334, had a vascular underlying factor. Of 204 male patients, 23 (11.3%) and out of 130 female patients, 8 (6.2%) had positive CTAs. (Table 4).

**Figure 3:**
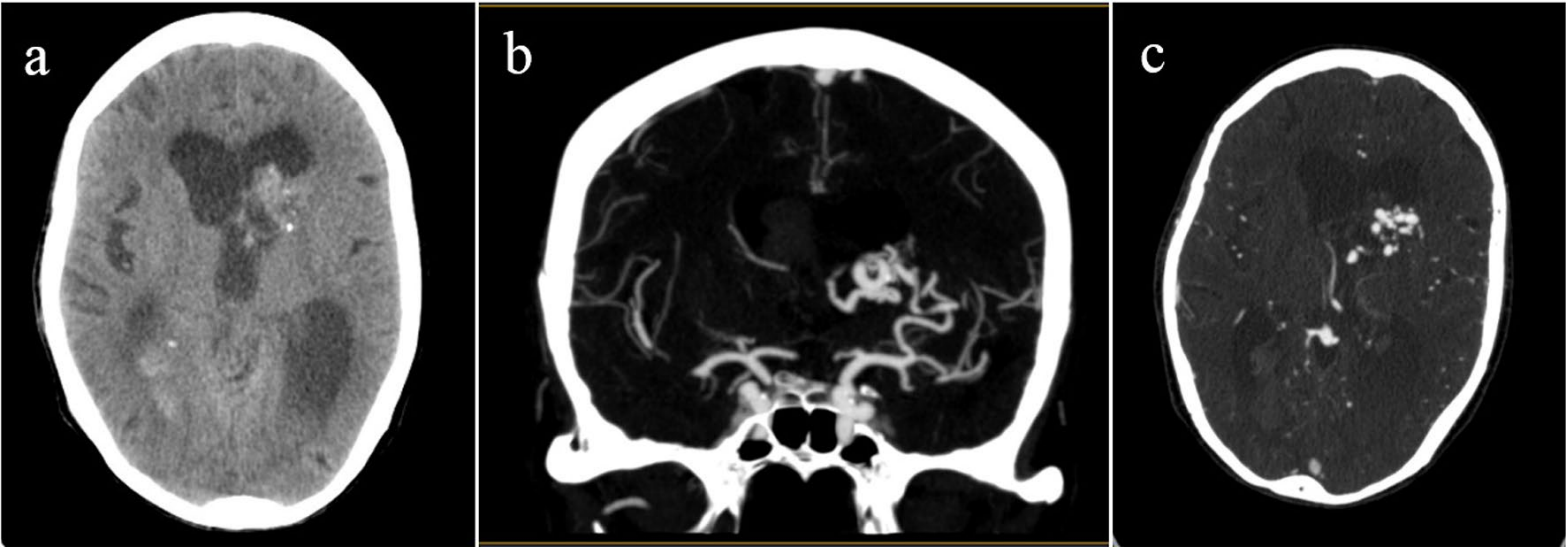
Non contrast CT scan (**a**) shows hyperdense lesion with small foci of calcification in the left caudate nucleus and intraventricular hemorrhage (IVH) in posterior horns of lateral ventricles. Coronal (**b**) and axial (**c**) cuts of brain CTA shows left caudate nucleus arteriovenous malformation (AVM) with feeding artery mostly from middle cerebral artery (MCA)insular branches and draining into deep veins.

**Figure 4:**
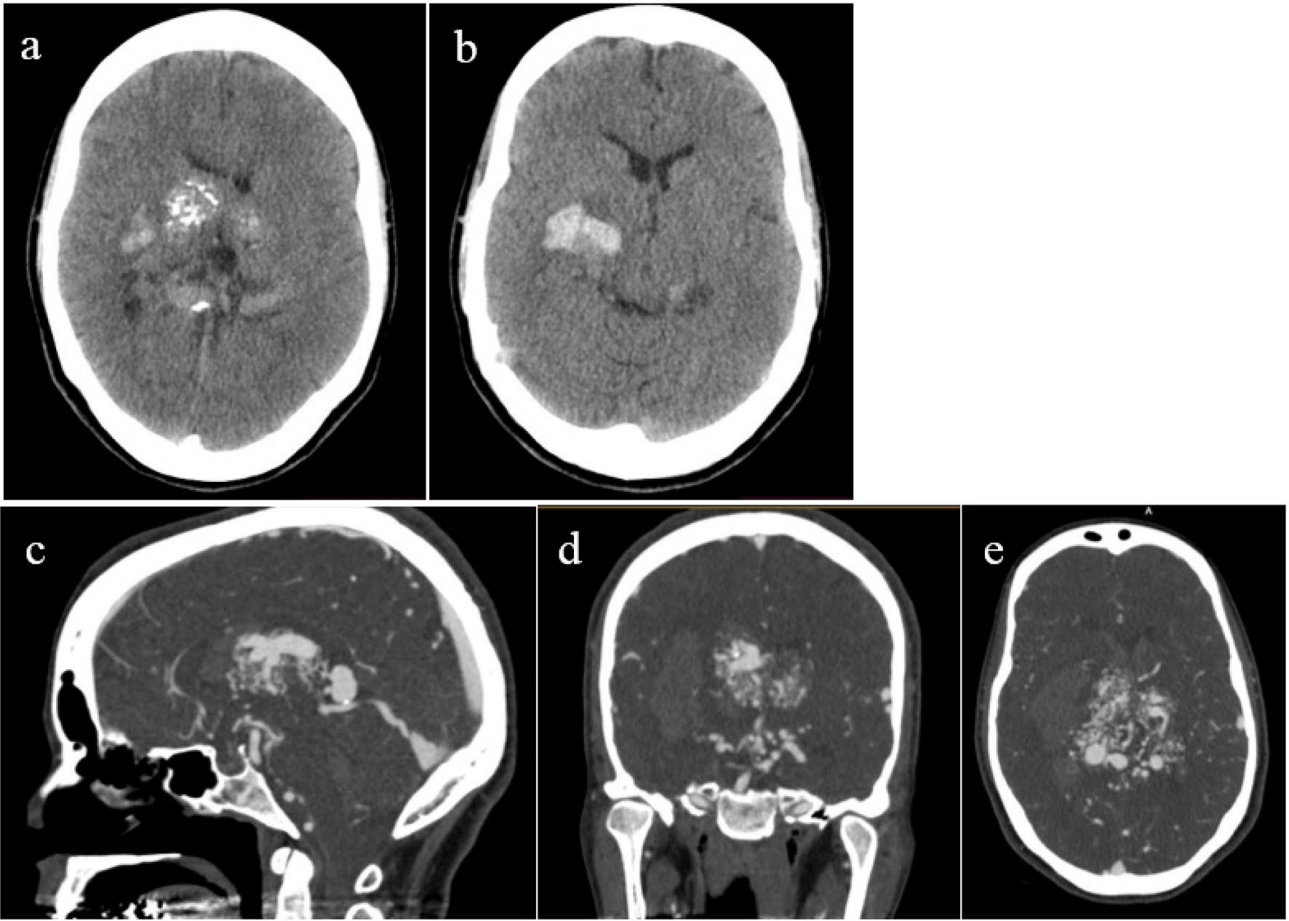
Axial non contrast CT scan show hyperdense lesion with multiple small foci of calcifications in bilateral basal ganglia and hemorrhage in right basal ganglia (**a**, **b**). Sagittal (**c**), coronal (**d**) and axial (**e**) cuts of brain CTA show large arteriovenous malformation (AVM) with feeding artery mostly from posterior circulation and draining mostly into the vein of Galen and deep veins.

The most common vascular etiology was intracranial AVM seen in 20 (6%) patients (Figs. 3 and 4). Table 2 summarizes the frequency of the different vascular ICH etiologies.

**Table 2:** Vascular etiologies of the spontaneous intracranial hemorrhage in 385 patients.

Etiology	Number of patients (%)
Non-vascular	304 (91)
AVM	20 (6)
Dural vein thrombosis	5 (1.5)
dAVF	2 (.6)
Deep vein anomaly	2 (.6)
Cavernoma	1 (.3)

AVM, arteriovenous Malformation; dAVF, dural arteriovenous fistulas.

### NCCT categorization

The interobserver agreement between the two radiologists for the NCCT categorization was 0.73, which is acceptable.

Of the 334 patients, 178 were categorized as low probability of the presence of an underlying vascular etiology (53.3%), and 10 were categorized as high probability of the presence of an underlying vascular etiology (3%). The remaining 146 (43.7%) NCCTs were categorized as indeterminate for the presence of an underlying vascular etiology. Of the 10 high probability NCCT examinations, 6 (60%) were true-positive and 4 (40%) were false-positive. Of the 178 low probability NCCT examinations, 170 (95.5%) were true-negative and 14 (4.5%) were false-negative. Hence, for the prediction of a vascular etiology for the ICH, high- and low probability NCCT examinations demonstrated a positive predictive value of 60%, and a negative predictive value of 95.5%, respectively. However, 17 of the 146 indeterminate NCCTs had an underlying vascular etiology for the ICH (11.6%). The results are summarized in Table 3.

**Table 3:** Distribution of Multidetector Computed tomography angiography (MDCTA) findings according to the Non-Contrast Computed tomography (NCCT) probability.

		Non vascular	AVM	CVT	dAVF	DVA	Total
NCCT category							
Low	Count (%)	171 (96.1)	7 (3.9)	0 (0)	0 (0)	0 (0)	178 (100)
Intermediate	Count (%)	129 (88.4)	11 (7.5)	2 (1.4)	2 (1.4)	2 (1.4)	146 (100)
High	Count (%)	4 (40)	2 (20)	3 (30)	1 (10)	0 (0)	10 (100)
Total	Count (%)	304 (91)	20 (6)	5 (1.5)	3 (0.9)	2 (0.6)	334 (100)

AVM, arteriovenous Malformation; dAVF, dural arteriovenous fistula; CVT, cerebral venous thrombosis; DVA, developmental venous anomaly.

### Clinical and radiologic predictors of a vascular ICH etiology

The results of univariate and multiple variables logistic regression analysis for the diagnostic yield of NCCT in the entire patient population, patients with lobar ICH, and the 2 different patient age groups are summarized in Table 4. The independent predictors of vascular etiology of ICH were age, known history of hypertension, location of hemorrhage, and presence of considerable edema adjacent to the lesion (Table 4).

**Table 4:** Univariate and multivariate regression analysis for the predictors of vascular etiology of Intracerebral hemorrhage (ICH).

		Number of patients (%)	Positive CTAs (%)	P value	
				Univariate analysis*	Multivariate analysis**
All patients		334 (100)	31 (9.3)	N/A	
Sex	Male	204 (61.1)	23 (11.3)	0.13	
	Female	130 (38.9)	8 (6.2)		
Age Group	1: 18–45 years	91 (27.2)	18 (19.8)	< 0.000	0.002
	2: > 45 years	243 (72.8)	13 (5.3)		
Known HTN	Yes	230 (68.9)	4 (1.7)	< 0.000	< .000
	No	104 (31.1)	27 (26)		
Impaired coagulation***	Yes	12 (3.6)	0 (0)	0.61	
	No	321 (96.4)	31 (9.7)		
Hemorrhage site	Lobar	119 (35.6)	21 (17.6)	0.002	0.009
	Deep grey matter or pons	170 (50.9)	8 (4.7)		
	Infratentorial	20 (6)	1 (5)		
	Multicompartment	25 (7.5)	1 (4)		
IVH	Yes	154 (46.1)	19 (12.3)	0.09	
	No	180 (53.9)	12 (6.7)		
SAH	Yes	35 (10.5)	6 (17.1)	0.29	
	No	299 (89.5)	25 (8.4)		
SDH	Yes	7 (2.1)	2 (28.6)	0.13	
	No	327 (97.9)	29 (8.9)		
Considerable edema	Yes	15 (4.5)	8 (53.3)	< 0.000	< 0.000
	No	319 (95.5)	23 (7.2)		

*Univariate analysis with Pearson X2 test, **Logistic regression model; CTA: Computed Tomography Angiography; HTN: hypertension; IVH: intraventricular hemorrhage; SAH: subarachnoid hemorrhage; SDH: subdural hemorrhage, *** There was 1 missing data.

### Vascular ICH (VICH) score

We used the independent predictors of a positive CTA, to construct a practical scoring system to predict the risk of vascular etiology in ICH patients (Table 5). The results of the application of this scoring system to the patient population are shown in Table 6. The diagnostic results of NCCT categorization and VICH score are compared in Table 7.

**Table 5:** calculation of Vascular Intracerebral hemorrhage (VICH) score.

Parameter		Points
NCCT categorization	High probability	2
	Indeterminate	1
	Low probability	0
Age group	18–45	1
	> 45	0
No hypertension	Yes	1
	No	0
Lobar hemorrhage	Yes	1
	No	0
Associated edema	Yes	1
	No	0

NCCT: Non-Contrast Computed tomography; IVH: intraventricular hemorrhage; SAH: subarachnoid hemorrhage.

**Table 6:** Predictive value of the Vascular Intracerebral hemorrhage (VICH) score.

Score	No. (%)	No positive CTAs (%)
0	108 (32.3)	1 (0.9)
1	71 (21.3)	1 (1.4)
2	73 (21.9)	4 (5.5)
3	55 (16.5)	9 (16.4)
4	22 (6.6)	13 (59.1)
5	5 (1.5)	3 (60)
6	0 (0)	NA
**ROC curve analysis**		
AUC (95% CI)	0.88 (0.81–0.94)	
Maximum operating point	> 4	
Sensitivity	51.6%	
Specificity	96.4%	
Accuracy	92.2%	
PPV	59.3%	
NPV	95.1%	

CTA, computed tomography angiography; ROC curve, receiver operating characteristic curve; AUC, area under the curve; CI, confidence interval; PPV, positive predictive value; NPV, negative predictive value.

**Table 7:** Comparison of the diagnostic accuracy of Non-Contrast Computed tomography (NCCT) categorization and Vascular Intracerebral hemorrhage (VICH) score.

	Positive threshold	Sensitivity	Specificity	Accuracy	NPV	PPV
NCCT categorization	Intermediate and high probability	**74.2**	56.1	57.8	**95.5**	14.7
	High probability	19.3	**98.7**	91.3	92.3	**60.0**
VICH score	> 4	51.6	96.4	**92.2**	95.1	59.3

Significant values are in bold.

## Discussion

Primary ICH constitutes 10–15% of cases of acute stroke. Although most cases of primary ICH are secondary to hypertension or coagulopathy, many cases have an underlying vascular etiology like AVF or intracranial aneurysm. Correct diagnosis of vascular etiologies is of utmost importance in the timely diagnosis and prevention of episodes of rebleeding.

In our study with a random selection of patients, 61.1% were male and 38.9% were female. The rate of detection of vascular findings in our patient cohort was 9.3% which is roughly similar to other studies. The number of positive CTAs was 11.3% in men and 6.2% in women. These figures were not statistically different (p: 0.13, chi-square test).

Cranial CTA is now accepted as the diagnostic standard for vascular etiologies of ICH. This modality is readily available in most centers and can be performed within seconds. However, the radiation exposure and risks of contrast nephropathy are two drawbacks of this imaging modality^11^, and it's good to use the NCCT as a guide to predict who may benefit more from cranial CTA^10^.

In the current study, we retrospectively evaluated the NCCT categorization previously described by Delgado *et al*.^10^ as a predictor of vascular etiology in patients with primary ICH. We showed that NCCT categorization had a positive and negative predictive value of 60% and 95.5% to predict the vascular causes of ICH. The results were similar to another study conducted by Almandoz et al. which reported the PPV and NPV of the NCCT to be 84.4% and 98% respectively^15^. In terms of the independent factors associated with a vascular etiology, we showed that lack of hypertension and coagulopathy, age of 45 years and younger, lobar location of hemorrhage, and presence of significant edema around the lesion were associated with higher risks of harboring a vascular etiology. Our study results agree with those of most previous conventional angiographic studies^12,13,16–18^.

We also developed a scoring system that predicts a given ICH patient’s risk of harboring an underlying vascular etiology based on the dependent factors associated with the presence of a vascular etiology (VICH score). The practicality of the VICH score lies in its ease of calculation because it requires only a review of the NCCT according to the NCCT categorization, location of hemorrhage, perilesional edema, and clinical data routinely obtained on presentation to the emergency department (patient age and history of hypertension).

**Figure 5:**
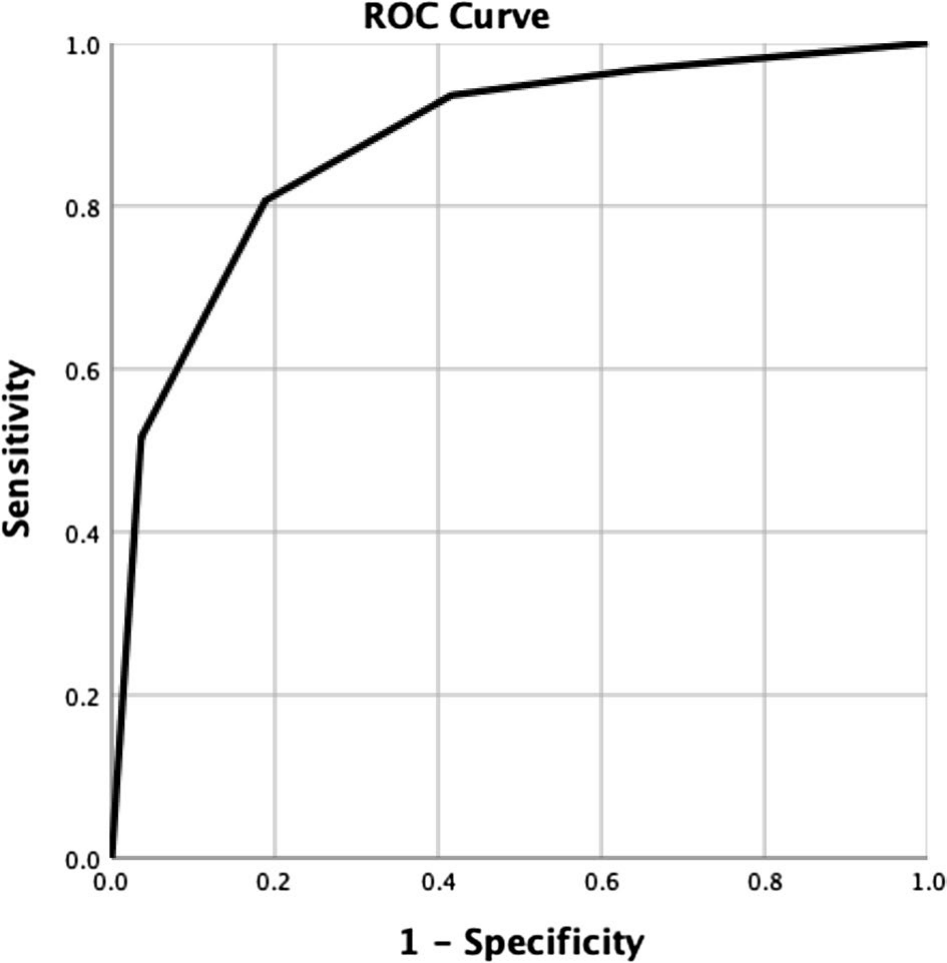
ROC curve analysis of the diagnostic accuracy of VICH score for predicting vascular etiology of intracranial hemorrhage.

In our study, one of the patients with a VICH score of 0 had a vascular etiology of ICH (0.9%), and this figure was 1.4% and 5.5% for the VICH scores of 1 and 2 respectively. This finding is very promising, especially in centers where neurovascular evaluation is not readily available, and could serve as a valuable tool to choose the patients who are most likely to harbor an underlying vascular abnormality. The ROC curve analysis showed that the VICH score ≥ 4 has a sensitivity and specificity of 51.6% and 96.4% respectively, which might be the maximum reasonable cut-off. (Fig. 5).

Many medical centers consider a positive rate of more than 10% for a low-risk noninvasive diagnostic tool such as MDCTA to be high enough to merit performing it in all patients^10^.

The limitations of our study are the retrospective nature of the study, the potential selection bias generated by the inclusion of only patients who presented with ICH and were evaluated with MDCTA, and the lack of independent validation of this scoring system. This scoring system might be helpful when there are limited resources, at the cost of missing some patients with possible vascular etiology.

## Conclusion

In this retrospective cohort of 334 patients, we found that the VICH score can be practical in institutions with limited resources to increase the sensitivity of finding an underlying vascular etiology. Further prospective studies are warranted to validated this scoring system in patients with primary ICH.

## Data Availability

The datasets generated during and/or analyzed during the current study are available from the corresponding author on reasonable request.
